# Electrodeposition of α-MnO_2_/γ-MnO_2_ on Carbon Nanotube for Yarn Supercapacitor

**DOI:** 10.1038/s41598-019-47744-x

**Published:** 2019-08-02

**Authors:** Jae-Hun Jeong, Jong Woo Park, Duck Weon Lee, Ray H. Baughman, Seon Jeong Kim

**Affiliations:** 1 Center for Self-powered Actuation, Department of Biomedical Engineering, Hanyang University, Seoul, 04763 Korea; 2 Department of Chemistry and Material Science, Aalto University, PO Box 16100, FI-00076 Aalto, Finland; 3 The Alan G. MacDiarmid NanoTech Institute, University of Texas at Dallas, Richardson, Texas 75083 USA

**Keywords:** Materials for energy and catalysis, Electrical and electronic engineering

## Abstract

Yarn supercapacitors have attracted renewed interest as promising energy storage for wearable devices due to their lightweight, long cycling lifetime and excellent weavability. There has been much effort to fabricate high performance yarn supercapacitor by depositing pseudo-capacitive materials on the outer surface of the carbon fibers. However, a key challenge still remains to achieve high capacitance and high mass loading without sacrificing the cycling stability. Herein, we perform a phase-controlled of MnO_2_ at various deposition temperatures with ultrahigh mass loading of 11 mg/cm^2^ on a MWNT sheets and fabricate it to yarn structure to achieve high capacitance without decreasing in the electrochemical performance. The structure of optimized sample (MnO_2_/CNTs-60, deposition at 60 °C) consists of the composite of primary α-MnO_2_ nanosheets and secondary γ-MnO_2_ nanoparticles. The heteronanostructures of MnO_2_ provide facile ionic and electric transport in the yarn electrode, resulting in improvement of electrochemical performance and cycling stability. The MnO_2_/CNTs-60 yarn electrode with ultrahigh mass loading delivers a high areal capacitance of 3.54 F/cm^2^ at 1 mA/cm^2^ and an excellent rate capability. Finally, the MnO_2_/CNTs-60 device exhibits an outstanding high areal energy density of 93.8 μWh/cm^2^ at the power density of 193 μW/cm^2^, which is superior to previously reported symmetric yarn supercapacitors.

## Introduction

With the rapid development of portable devices and wearable electronics, the yarn supercapacitors has been continuously demanded because of their high power density, lightweight, long cycling lifetime and excellent weavability^1–3^. The multiwalled carbon nanotubes (MWNTs) as electrode materials has been utilized in yarn supercapacitors due to its high surface area, good mechanical strength, flexibility and excellent electrical conductivity^4–7^. However, the MWNTs yarn supercapacitors have several urgent disadvantages such as low specific capacitance and low energy density, leading to seriously suffering from their practical applications. Recently, the pseudocapacitive-type electrode materials have gained much attention due to getting the high capacitance by the charge stored through ion adsorption and surface redox reactions. Among various materials, manganese oxide (MnO_2_) is a promising material because of the abundant resources, low fabrication cost, and high theoretical capacitance^8–10^. More importantly, it has a wide potential window in a neutral aqueous electrolyte and therefore can achieve higher energy density than other cathode materials such as NiO, Ni(OH)_2_, Ni-Co and PANI^11–16^. However, the using a solely single phase MnO_2_ as electrode for supercapacitors due to some inherent disadvantages such as poor electrical conductivity and slow ion transport rate is poor in low rate capacity and cycle stability^17,18^. In order to overcome the drawbacks of MnO_2_, the co-existence of two-phase MnO_2_ materials exhibiting improved electrochemical performance due to synergy effect is one of the promising solutions^18,19^.

The fabrication of MnO_2_ on the MWNTs yarn through the electrodeposition is one of the important strategies to improve the capacitance of the MWNTs fiber-based supercapacitors^20–23^. Up to now, however, when an electrode is produced by the electrodeposition method in a yarn supercapacitor, the MnO_2_ are directly electrodeposited on the yarn electrode, so that the acceptable load of the MnO_2_ is limited. In several reported papers, the active material was electrodeposited on twisted CNT yarns and CNT coated spiral nylon fibers used as the core structure, wherein the amount of active material was limited to less than 20 wt%^20–22^. Therefore, a small active material loading exhibits low capacitance and energy stored, which restrict their practical application for high energy systems^24,25^. Generally, to provide a feasible energy for commercial devices, the high active loading of 8–10 mg/cm^2^ is required^26,27^. However, the increase in the loading active material significantly reduces the charge storage capacity, including specific capacitances and rate performance because of the low electrical conductivity, slow ion diffusion and poor mechanical stability of the MnO_2_ active material.

Herein, to overcome the aforementioned drawback and achieve both high capacitance and loading, the MnO_2_ was directly deposited on the MWNTs sheets through the electrodeposition technique, and then it was fabricated to yarm structure using biscrolling method. By depositing MnO_2_ onto MWNTs sheets, it dramatically expands the loading of active materials in yarn to as high as 11 mg/cm^2^. The MnO_2_ material composed of primary α-MnO_2_ nanosheets and secondary γ-MnO_2_ nanoparticles was grown on the surface of MWNTs sheets using an electrodeposition method at the different deposition temperature. Among them, the MnO_2_/CNTs-60 yarn electrode exhibits excellent areal capacitance of 3.54 F/cm^2^ at 1 mA/cm^2^. It is one of the highest values reported for MnO_2_-based yarn supercapacitors in gel electrolytes. In addition, it avoids the problem of general mechanical separation of composite materials during long-term cycling, and can improve the cycling stability. The MnO_2_/CNTs-60 device shows high areal energy density of 93.8 μWh/cm^2^ at the power density of 193 μW/cm^2^. This performance is the highest value in the most of the symmetric yarn supercapacitors.

## Results and Discussion

**Figure 1:**
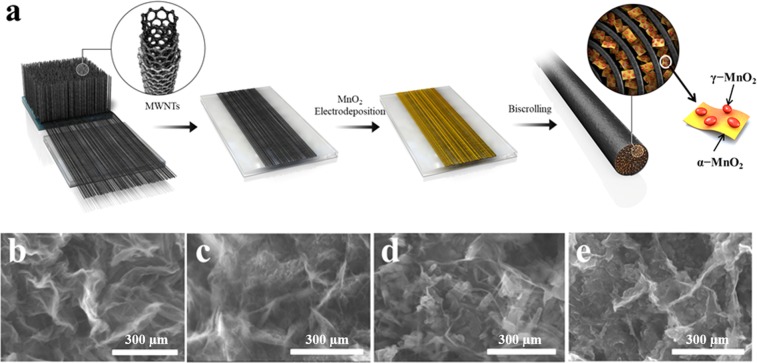
(**a**) Overview schematic illustrations showing the fabrication processes of yarn supercapacitor. The SEM images of morphology of the MnO_2_/CNTs yarn electrode with different deposition temperature: (**b**) MnO_2_/CNTs-25, (**c**) MnO_2_/CNTs-40, (**d**) MnO_2_/CNTs-60 and (**e**) MnO_2_/CNTs-80 yarn electrodes with around 96 wt% MnO_2_ particles. (scale bar = 300 nm).

A schematic illustration of the fabrication process for the yarn supercapacitor is presented in Fig. 1a. The five layers of MWNT sheets were stacked on a glass slide. Subsequently, the stacked MWNT sheets were immersed into a 0.1 M Mn(CH_3_CO_2_)_2_.(H_2_O)_n_ aqueous solution for 40 mins. After deposition, the MnO_2_/MWNT hybrid sheets were washed with ethanol/water (volume ratio of 1:1). The MnO_2_/MWNT hybrid sheets were peeled off from the glass slide and then twisted to form yarn supercapacitor through an electric motor.

The MnO_2_ was directly deposited on the MWNTs sheets through the electrodeposition at different temperatures and its morphologies of the all yarn samples, as presented in Figs 1b–e and S1, were observed through the SEM. At 25 °C of deposition temperature, interconnected MnO_2_ nanosheets grown on the surface of the MWNTs sheets are shown in Fig. 1b (MnO_2_/CNTs-25). When the deposition temperature increases at 40, 60 and 80 °C, respectively, it can be seen that not only similar sheets are observed but also small particles are on the nanosheets (MnO_2_/CNTs-40, 60 and 80, respectively, Fig. 1c–e). The nanosheets are preferred as primary structure to grow on the MWNTs sheets at the early stages of electrodeposition, but the morphologies of secondary particles in the MnO_2_/CNTs yarn depend on the deposition temperature. Conversely, at 25 °C, the growth of the primary nanosheets is predominant and secondary morphology is not observed. This is because more nucleation sites are allowed to occur on the surface of the nanosheets at the increase in the temperature.

**Figure 2:**
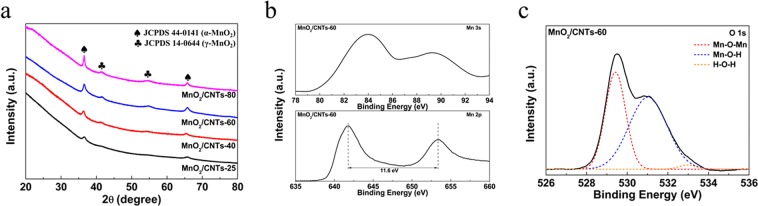
(**a**) XRD patterns of the MnO_2_/CNTs-25, MnO_2_/CNTs-40, MnO_2_/CNTs-60 and MnO_2_/CNTs-80 yarn electrodes. (**b**) Mn 2p and Mn 3 s XPS spectra and (**c**) the specific fitting of the O 1 s XPS peaks of the MnO_2_/CNTs-60 yarn electrode.

The crystal structure of the electrodeposition MnO_2_ is investigated by X-ray diffraction (XRD) and shown in Fig. 2(a). The two characteristic peaks of MnO_2_/CNT-25 yarn electrode at the diffraction angle 2θ = 37.5°, 65.5° are indexed to the (211) and (002) of the α-MnO_2_ phase (JCPDS 44-0141). The intensity of diffraction peaks is broaden, indicating the poor crystallinity of α-MnO_2_ in the composite. When the deposition temperature increases from 40 °C to 80 °C, there is not only the α-MnO_2_ phase, but also two diffraction peaks corresponding to the γ-MnO_2_ at 2θ = 42.1° and 55.5° (JCPDS 14-0644), which are assigned to the (300) and (160) crystal plane. This indicates that the α-MnO_2_ phase nanosheets was initially grown on the MWNTs sheets, while the nanoparticles with γ-MnO_2_ phase were secondarily grown from the deposition temperature of 40 °C, which is consistent with the SEM results. With the increase in the electrodeposition temperature up to 80 °C, the intensity of diffraction peaks of α-MnO_2_ phase is sharper, indicating high crystallinity of α-MnO_2_ phase compared to the other samples. Moreover, the diffraction peaks of γ-MnO_2_ phase for MnO_2_/CNT-80 yarn electrode are clearly observed, indicating that the large amount of γ-MnO_2_ phase is formed compared to the other samples. Overall, as the deposition temperature increases, the main crystalline phase of samples has changed from a pure α-MnO_2_ into a mixture of α-MnO_2_ and γ-MnO_2_.

All samples were investigated by X-ray photoelectron spectroscopy (XPS). The Mn and O elemental spectra of the MnO_2_/CNTs-60 sample are shown in Fig. 2b,c and the other samples are present in Figs S2–S5. On the basis of the analysis of the Mn 2p spectrum, the characteristic peaks at 641.7 and 653.3 eV correspond to the Mn 2p_1/2_ and Mn 2p_3/2_ spin-orbit peaks. The spin-energy separation of two peaks is 11.6 eV, which is in good accordance with previously reported values for the MnO_2_ materials^28–30^. In the Mn 3 s spectrum, the binding energy separation of the two peaks for Mn 3 s means an average oxidation state of Mn of MnO_2_^28,29^. According to previous reports, the separation value of 4.7 eV and 5.4 eV corresponds to Mn^4+^ and Mn^3+^^29,30^. The binding energy separation is 5.2 for MnO_2_/CNTs-25, 5.2 for MnO_2_/CNTs-40, 5.1 for MnO_2_/CNTs-60, and 4.9 for MnO_2_/CNTs-80, respectively, which suggests an intermediate oxidation state peak between Mn^4+^ and Mn^3+^. This means that the deviation from Mn^4+^ is a result of the formation of defects during the electrodeposition process. Finally, the oxidation states of Mn in MnO_2_ were estimated by the O 1s peak. The O 1s peaks are deconvoluted with three components, representing the Mn-O-Mn component at 530.2 eV, Mn-O-H component at 531.5 eV, and the H-O-H at 532.6 eV (Figs 2c and S8). The valence of Mn can be also calculated to be 3.42 through the intensities ratio of the Mn-O-Mn and Mn-OH according to a previous study. This result is in good agreement with the XPS analysis of the Mn 3s spectrum^31^.

**Figure 3:**
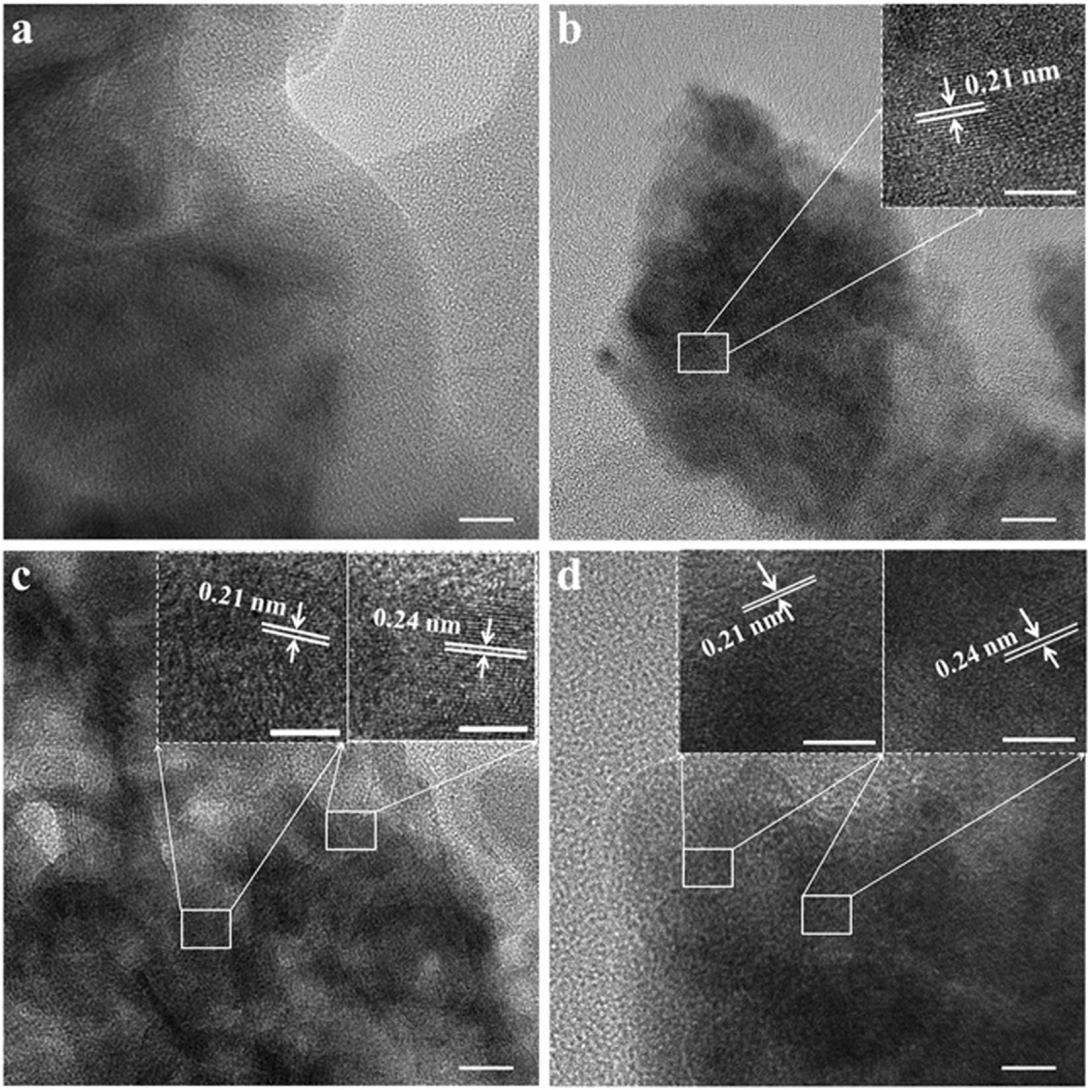
The TEM images of MnO_2_ particles for (**a**) MnO_2_/CNTs-25, (**b**) MnO_2_/CNTs-40, (**c**) MnO_2_/CNTs-60 and (**d**) MnO_2_/CNTs-80 yarn electrodes. (scale bar = 10 nm) The insets of figures show the high resolution TEM images. (scale bar = 5 nm).

In order to confirm the two phases in the MnO_2_/CNTs yarn electrodes, transmission electron microscopy (TEM) characterization was conducted. Figure 3a displays the α-MnO_2_ nanosheets with amorphous structure in the MnO_2_/CNTs-25 sample. In the case of MnO_2_/CNTs-40 electrode, similar large particles corresponding to the amorphous of α-MnO_2_ are observed at low magnification TEM image (Fig. 3b), as well, the small particles with orderly lattice planes can be clearly observed in the inset of Fig. 3b. The orderly lattice planes are assigned to the (300) plane (d = 0.21 nm) of γ-MnO_2_ crystal structure, confirming the existence of two types phases in the MnO_2_/CNTs-40 yarn electrode. Moreover, at higher temperatures, the amorphous nanosheets are basically present for the samples and it can be seen that the size of the particles with an orderly lattice plane increase. In the HRTEM images of the MnO_2_/CNTs-60 and 80 samples (Fig. 3c,d), γ-MnO_2_ present as well as there is other orderly lattice plane, which is indexed to the (211) plane (d = 0.24 nm) of α-MnO_2_ crystal structure. As mentioned in the XRD result, it is confirmed that the α-MnO_2_ crystal structure with high crystallinity appears. Meanwhile, the TEM element mapping shows the uniform distributions of Mn and O elements in the MnO_2_/CNTs-60 profile (Fig. S6). Hence, it is verified that the co-existence of two MnO_2_ phases is showed in the MnO_2_/CNTs-40, 60 and 80 samples.

**Figure 4:**
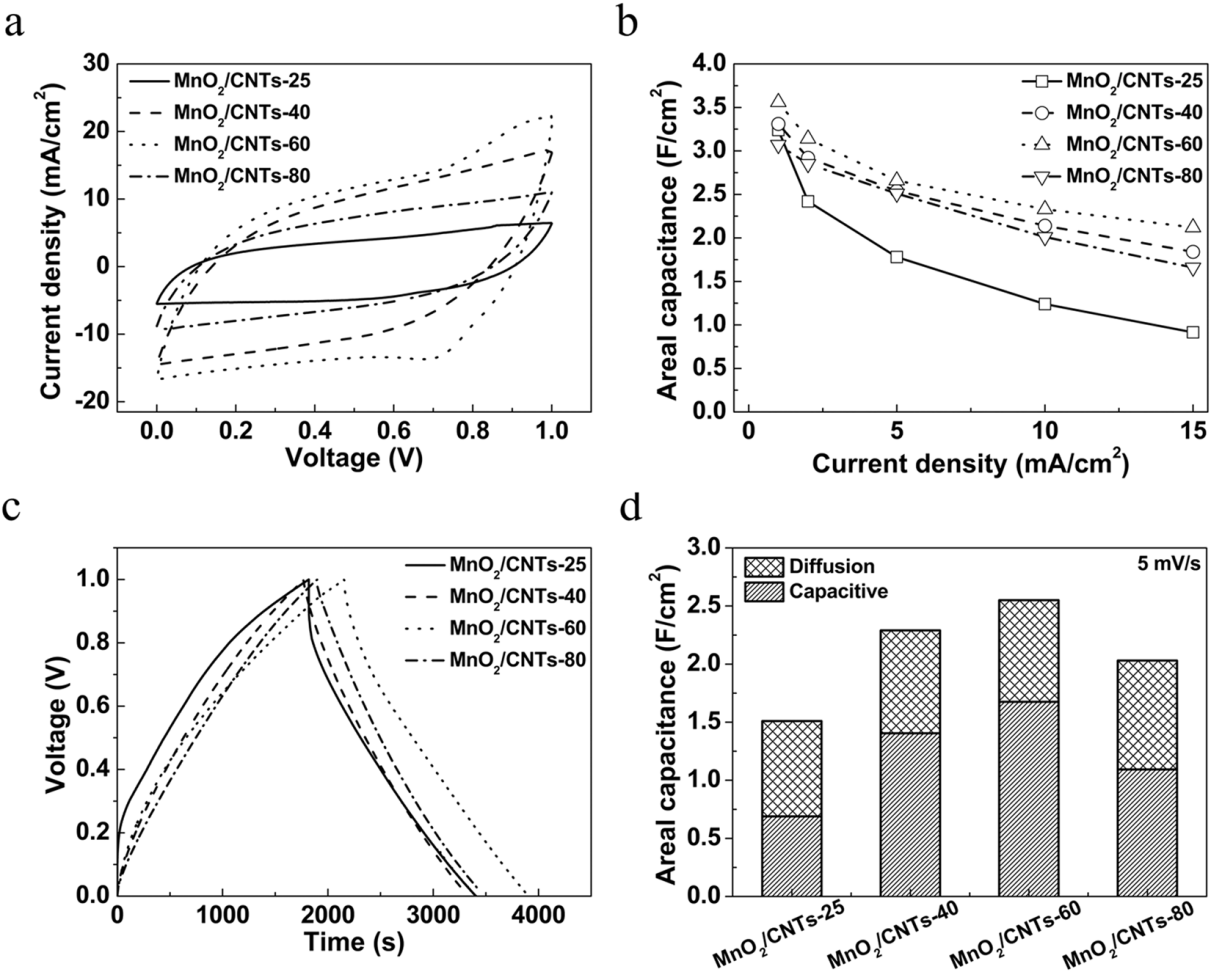
Electrochemical performance of the solid-state MnO_2_/CNTs-25, MnO_2_/CNTs-40, MnO_2_/CNTs-60 and MnO_2_/CNTs-80 yarn electrodes. (**a**) CV curves of the MnO_2_/CNTs-25, MnO_2_/CNTs-40, MnO_2_/CNTs-60 and MnO_2_/CNTs-80 yarn electrodes measured at a scan rate of 10 mV/s. (**b**) GCD profiles of the MnO_2_/CNTs-25, MnO_2_/CNTs-40, MnO_2_/CNTs-60 and MnO_2_/CNTs-80 yarn electrodes measured at 1 mA/cm^2^ (**c**) areal specific capacitance measured of each electrode at different current densities in the potential range of 0–1 V. (**d**) Capacitive and diffusive capacitance contribution at a scan rate of 5 mV/s.

The electrochemical performances were conducted for the MnO_2_/CNTs-25, MnO_2_/CNTs -40, MnO_2_/CNTs-60 and MnO_2_/CNTs-80 electrodes. Two electrodes cell was fabricated in parallel containing an aqueous poly(vinyl alcohol) (PVA)/LiCl gel electrolyte and then assembled to a solid-state yarn supercapacitor. Figure 4a shows the cyclic voltammetry (CV) curves of all samples at scan rate of 10 mV/s and CV curves of all samples at various scan rates are presented in Fig. S7. The quasi-rectangular shaped CV can be seen in all samples, indicating the energy storage by electrochemical double-layer charging capacitance of the CNTs and the pseudocapacitance of MnO_2_. As the deposition temperature increases up to 60 °C, the capacitance also increases. However, as the deposition temperature is further increased to 80 °C, the capacitance in the MnO_2_/CNTs-80 yarn electrode decreases. This phenomenon is also observed when the galvanostatic charge-discharge (GCD) curves of all samples were measured. Figure 4b represents the GCD profile of each electrode at the current density of 1 mA/cm^2^ and the results of measurement at different current densities (1,2,5,10 and 15 mA/cm^2^) are shown in Fig. S8. The weight, areal and volume capacitances of all samples with MnO_2_ loadings of 11 mg/cm^2^ are summarized in Table S1. The MnO_2_/CNTs-60 yarn electrode delivers the high areal capacitance of 3.56 F/cm^2^ at 1 mA/cm^2^, which is higher than the others yarn electrodes (for MnO_2_/CNTs-25, for MnO_2_/CNTs-40, for MnO_2_/CNTs-80). As previously aforementioned, the heterostructures would cause lattice defects between the intersection of two phases, leading to create electrochemical active sites and increase for fast electron transportation. In the case the MnO_2_/CNTs-80 yarn electrode, however, it has two phases, but the large particle with high crystallinity is the major drawback for its ionic and electronic conductivity in comparison to the MnO_2_/CNTs-40 and 60, resulting to slightly decrease in the electrochemical performance. The MnO_2_/CNTs-40, 60 and 80 yarn electrodes also exhibit excellent rate capability performance with capacitance retention of 55.6, 59.6 and 54.1%, respectively, when the current densities increase from 1 mA/cm^2^ to 15 mA/cm^2^, demonstrating the advantage of existence of two phases. In addition, it is hard to come off the MnO_2_ powder from MWNTs sheets because it is wrapped by the MWNTs sheets (Fig. S1). Therefore, the excellent rate capability is obtained due to the intrinsic nature of the heterophases and MWNTs of the MnO_2_/CNTs-40, 60 and 80 yarn electrodes. Moreover, in the Nyquist and electrical conductivity plots (Figs S9 and S10 in Supporting information), the MnO_2_/CNTs-60 yarn electrode shows the lowest equivalent series resistance (Rs) value and high electrical conductivity (50.5 S cm^−1^) compared with the others samples. This is because the MnO_2_/CNTs-60 yarn electrode has the high surface area and large reactive active sites compared with the others samples. As a result, the MnO_2_/CNTs-60 yarn electrode exhibits the excellent capacitance characteristic with fast electrolyte ion response. In the contrast, the areal capacitance of MnO_2_/CNTs-25 yarn electrode retained only 28.2% with the increase of current density. It is indicated that single phase MnO_2_ as electrodes suffers from low rate capacity due to high resistance and low electrical conductivity.

In our case, the two phases of MnO_2_ in the MnO_2_/CNTs composites provides the improvement of the electron transportation between electrode and electrolyte, leading to higher capacitive current than the one phase MnO_2_. To demonstrate this, the detailed charge storage mechanisms and electrode kinetics capacitances were calculated by Dunn’s method based on the CV curves at various scan rates^32,33^. The capacitance of all samples obtained from CV curves can be separated as the capacitive charge storage and the diffusion controlled insertion processes. The capacitive-controlled capacitances are 45.7% for MnO_2_/CNTs-25, 61.3% for MnO_2_/CNTs-40, 65.7% for MnO_2_/CNTs-60 and 53.8% for MnO_2_/CNTs-80, respectively. The high value of capacitive-controlled capacitance means that the charge storage process can be easily facilitated in the electrode and leads to its excellent rate capability. On the other hands, the low capacitive-controlled capacitances values in the other three electrodes indicate slower kinetics, resulting in the poor rate capability. Consequently, the low charge transfer resistance, small electrical resistance and high capacitive-controlled capacitances of MnO_2_/CNTs-60 yarn electrode establish inherently excellent electrochemical performance.

**Figure 5:**
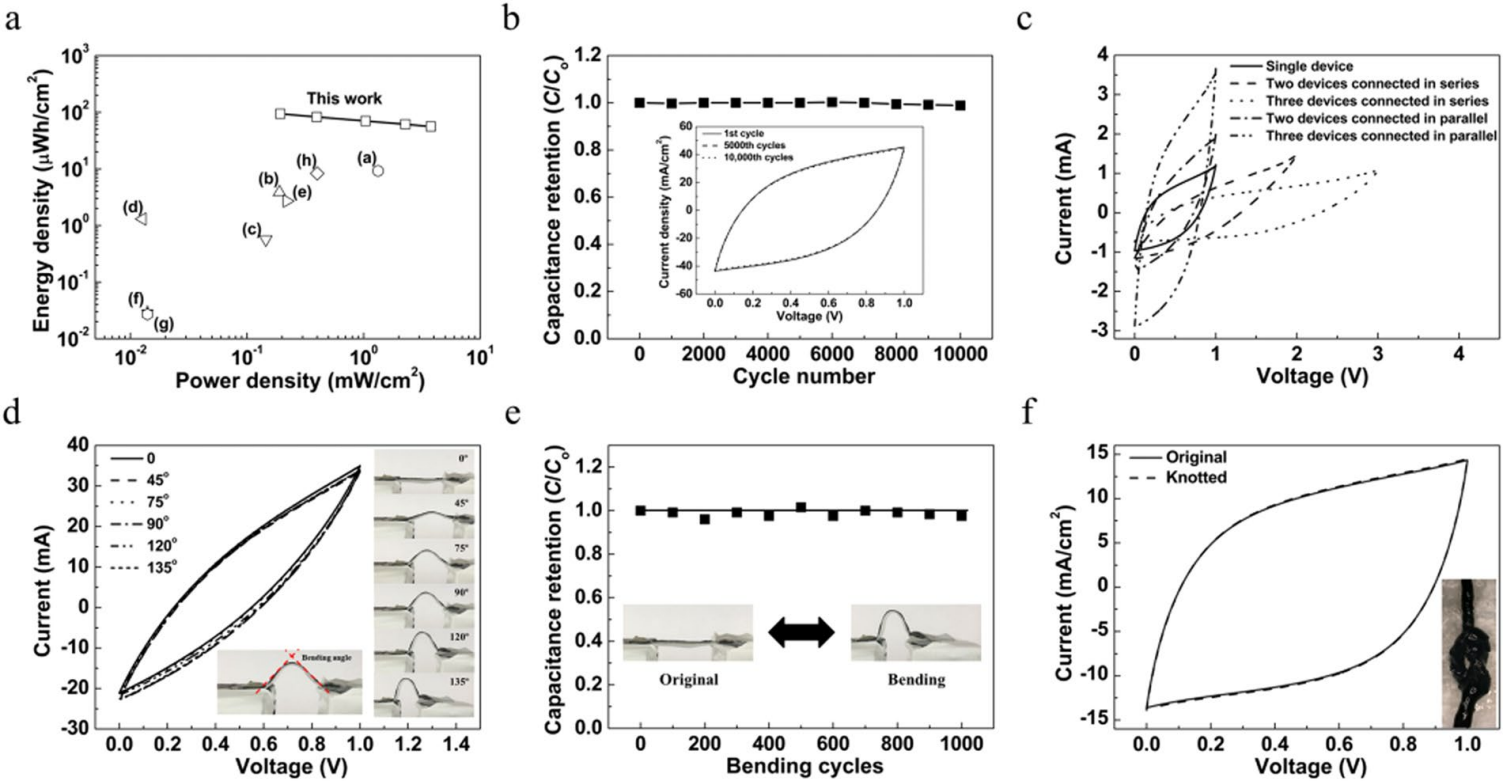
(**a**) The areal energy and power density of MnO_2_/CNTs-60 yarn electrode compared with those of previously published results. The maximum areal energy density of the MnO_2_/CNTs-60 yarn electrode is 93.8 μWh/cm^2^. This value is higher than the previously reported yarn supercapacitors, which contain (**a**) PPy/MnO_2_/rGO (9.2 μWh/cm^2^), (**b**) rGO/CNT (3.84 μWh/cm^2^), (**c**) PANI/CNT,(0.57 μWh/cm^2^), (**d**) MnO_2_/MPNW (1.3 μWh/cm^2^), (**e**) pen ink Au/plastic wire (2.7 μWh/cm^2^), (**f**) MnO_2_/ZnO (0.03 μWh/cm^2^), (**g**) ZnO nanowire (0.027 μWh/cm^2^), (**h**) PEDOT-S:PSS fiber (8.3 µWh/cm^2^) and (**i**) biscolled MnO_2_/CNT (35.8 μWh/cm^2^). (**b**) Cycle stability of MnO_2_/CNTs-60 yarn electrode under a scan rate of 50 mV/s as a function of cycle number. (**c**) CV curves of three connected in parallel and in series (scan rate = 50 mV/s). (**d**) CV curves of the MnO_2_/CNTs-60 supercapacitor under different bending angles at a scan rate of 50 mV/s. The right and bottom insets show the optical images of different bending angles and the optical image of bending at 90°, respectively. (**e**) Capacitance retention of the MnO_2_/CNTs-60 supercapacitor during the bending cycles. The inset shows optical images of pristine and bending state and the bending degree is 135°. (**f**) CV curves (at 30 mV/s) for the MnO_2_/CNTs-60 yarn electrode. The inset shows the optical image of a knotted the MnO_2_/CNTs-60 yarn electrode.

Figure 5a shows a Ragone plot of areal energy density versus power density compared with the previously reported supercapacitors. Based on the total surface area of the supercapacitor, including gel electrolyte, the areal energy density and power density of symmetric MnO_2_/CNTs-60 device was calculated. The maximum areal energy density was 93.8 μWh/cm^2^ at 193 μW/cm^2^, which is higher than previously published studies such as (a) PPy/MnO_2_/rGO, (b) rGO/CNT, (c) PANI/CNT, (d) MnO_2_/MPNW, (e) pen ink Au/plastic wire, (f) MnO_2_/ZnO, (g) ZnO nanowire, (h) PEDOT-S:PSS fiber (i) biscolled MnO_2_/CNT^34–42^. Figure 5b shows the capacitance retention of the symmetric MnO_2_/CNTs-60 device at a scan rate of 50 mV/s during 1000^th^ cycles. The symmetric MnO_2_/CNTs-60 device exhibits excellent cycling stability with 98.9% under 1000th cycles because it has a good flexibility by hetero morphologies of MnO_2_ and MWNT sheets. More importantly, this structure helps to buffer the internal deformation during cycling. In addition, these α-phase components stably maintain long-term cycling due to the large ion tunnels, and multiple junctions between the α- and γ-phases help to further buffer internal crystal deformation. These phenomenons ensure excellent mechanical stability which effectively inhibits electrode degradation and improves cycling stability. In order to demonstrate the practical application of the device and to meet the voltage or power requirements for practical applications, the MnO_2_/CNTs-60 devices are required to be connected in series or in parallel. As shown in Fig. 5c, the voltage window and current density increase when devices are connected in series and in parallel, respectively. The MnO_2_/CNTs-60 devices can operate a red light emitting diode (LED, 1.8–2.2 V) even bending. (Fig. S11 in the Supporting information) Moreover, to briefly demonstrate the ability to withstand harsh banding, the MnO_2_/CNTs-60 sample was measured under different bending angles from 0° to 135° at a scan rate of 50 mV/s. As illustrated in Fig. 5d, the changes in CV curves are negligible, indicating the outstanding flexibility of our devices. In addition, as shown in Fig. 5f, negligible change was observed even knotted. To investigate the stability after bending 1000 cycles, the capacitance retention was maintained after 1000 cycles of bending from 0° to 135°, demonstrating the robust mechanical property of our device. (Fig. 5e).

## Conclusion

A high mass loading of 11 mg/cm^2^ and the heterophases of MnO_2_ were deposited on MWNTs sheets through a facile electrodeposition technique, which was made of yarn electrode. When the deposition temperature increases, the α- and γ-phases of MnO_2_ in MnO_2_/CNTs can be obtained. The MnO_2_/CNTs-60 in optimized material is composed of α- and γ-phases of MnO_2_, which create electrochemical active sites and improve the fast electron transportation. The MnO_2_/CNTs-60 yarn electrode shows an extremely areal capacitance of 3.54 F/cm^2^ at 1 mA/cm^2^ in a gel electrolyte, which is superior to previously reported MnO_2_ yarn electrodes. Also, the MnO_2_/CNTs-60 yarn electrode has the good mechanical stability as well as high ionic and electric conductivities of the material due to the heterophases of MnO_2_ and wrapping of MnO_2_ particles by MWNT sheet, resulting that it shows excellent cycle retention capacitance with >98% during 1000 charge/discharge cycles. Significantly, the MnO_2_/CNTs-60 device delivers an extremely high areal energy density of 93.8 μWh/cm^2^ at the power density of 193 μW/cm^2^. Our results suggest that the heterostructures with high mass loading enhance the electrochemical performance. It will be the possibility to be applied in practical applications.

## Method

### Materials

Lithium chloride (LiCl, >99%), poly(vinyl alcohol) (PVA, M*w* 146,000~186,000) and manganese acetate (Mn(CH_3_CO_2_)_2_.(H_2_O)_n_) were purchased from Sigma-Aldrich.

### Electrodeposition of Manganese Oxide (MnO_2_) on aligned carbon nanotube sheets

As shown in Fig. 1a, the five layers of highly aligned carbon nanotube sheets with the width of ~2 cm and length of ∼7.5 cm which were drawn from the multiwalled nanotube (MWNT) forest (U053HANYANG-SH158-06, LINTEC Inc.) were stacked on the glass side^20,22,42^. Subsequently, the stacked MWNT sheets was immersed in a 0.1 M manganese acetate aqueous solution to do the electrodeposition of MnO_2_ on the MWNT sheets using a potentiostatic method. The electrodeposition of MnO_2_ on the stacked MWNT sheets was conducted at about 1.3 V for 40 mins using Ag/AgCl as a reference electrode and Pt mesh as a counter electrode in a three electrode system through an electrochemical analyzer (CHI 627b system, CH Instruments, Austin, TX). In order to investigate the effect of temperature on MnO_2_ growth on the stacked MWNT sheets, the electrodeposition of MnO_2_ was carried out at various temperature of 25, 40, 60, 80 °C. These samples were named as MnO_2_/CNTs-25, MnO_2_/CNTs-40, MnO_2_/CNTs-60, and MnO_2_/CNTs-80, respectively. After electrodeposition, all of samples were washed thoroughly by deionized water and then it was peeled off from glass slide and twisted to ~100 turns per meter using an electric motor to form a yarn electrode.

### Supercapacitor assembly

The capacitive performance of solid-state yarn supercapacitor was measured through a two-electrode system. The device was fabricated by placing two MnO_2_/CNTs yarns in parallel, and then coating the PVA-LiCl (6 M) gel electrolyte. The 3 g of PVA and 6 g LiCl was dissolved in 30 ml deionized water at 90 °C for several hours to prepare the PVA/LiCl gel electrolyte. The Cu wires were attached at the end of two yarns using Ag paste for electrochemical performance measurement.

### Calculation of electrochemical performance

The capacitances of two electrode configuration were calculated from galvanostatic charge-discharge curve by following equation, C = I/(*dV*/*dt*) where, I and dV/dt are the discharge current and the slope of the discharge curve, respectively. The specific capacitance of the electrode was calculated by C*s* = C/S, where S is area (a), volume (v) and mass (g) of the yarn. The length of the yarn electrodes was fixed to 1 cm. In case of the two electrode systems, area and volume contain both electrodes and the PVA/LiCl gel electrolyte. The specific energy density and power density were calculated from the equation ${E}_{s}=\frac{1}{3600}\frac{1}{2}{C}_{s}{\rm{\Delta }}{V}^{2}$ and ${P}_{s}=\frac{{E}_{s}}{{\rm{\Delta }}t}$, where Δt is the discharging time.

### Characterization

The surface morphologies of the materials were observed using a scanning electron microscope (SEM, Hitachi S-4800, Japan). Transmission electron spectroscopy (TEM) images were taken with JEOL-2100F at an acceleration voltage of 200 kV. To determine the mass loading of MnO_2_ in the MnO_2_/CNTs yarns, the weight difference of the electrode was measured before and after electrodeposition using a Meter Toledo XP2U semi-microbalance with a readability of 1 μg. The crystal structures of the samples were investigated by X-ray diffraction (XRD, SmartLab, Rigaku). X-ray photoelectron spectroscopy analyses were carried out with Al K*α* radiation (XPS, K-alpha plus, Thermo Scientific, USA). All XPS spectra were calibrated using C 1s photoelectron peak at 284.6 eV as the reference. The electrochemical performances of the MnO_2_/CNTs yarns were obtained by a CHI 660E electrochemical workstation. Electrochemical impedance spectra (EIS) were conducted by applying a sinusoidal voltage of 5 mV in a frequency range from 0.01 to 100 kHz.

## Supplementary information

Supporting Information
