# Supplementary material for: Electrodeposition of α-MnO2/γ-MnO2 on Carbon Nanotube for Yarn Supercapacitor
Source: Sci Rep. 2019 Aug 2;9:11271. doi: 10.1038/s41598-019-47744-x (PMC6677808; doi:10.1038/s41598-019-47744-x)
Supplement: Supplementary file 1 — Supporting Information [file 41598_2019_47744_MOESM1_ESM.docx]

**(Supporting Information)**

Electrodeposition of α−MnO_2_/γ−MnO_2_ on Carbon Nanotube for Yarn Supercapacitor

**Jae-Hun Jeong^1^, Jong Woo Park^1^, Duck Weon Lee^1,2^, Ray H. Baughman^3^ and Seon Jeong Kim^1*^**

1. J.-H. Jeong, J. W. Park, D. W. Lee, and Prof. S. J. Kim^*^

Center for Self-powered Actuation, Department of Biomedical Engineering, Hanyang University, Seoul 04763, Korea

2. D. W. Lee

Department of Chemistry and Material Science, Aalto University, PO Box 16100, FI-00076 AALTO, Finland

3. R. H. Baughman

The Alan G. MacDiarmid NanoTech Institute, University of Texas at Dallas, Richardson, Texas 75083, USA.

*To whom correspondence should be addressed. E-mail: [sjk@hanyang.ac.kr](mailto:sjk@hanyang.ac.kr) (S.J.K)

**KEYWORDS:** manganese oxide, carbon nanotube, multi phases, high mass loading, supercapacitor


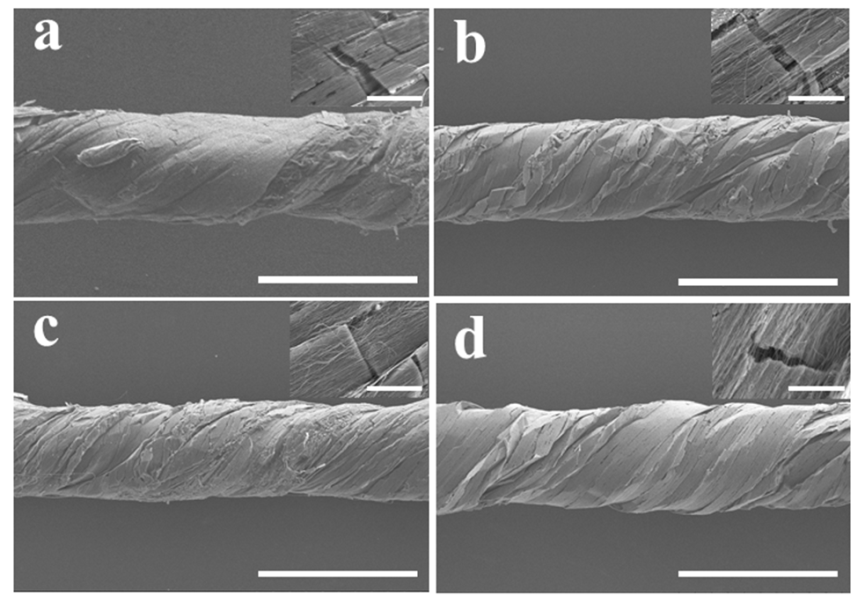


**Figure S1.** The SEM images of the (a) MnO_2_/CNTs−25, (b) MnO_2_/CNTs−40, (C) MnO_2_/CNTs−60 and (d) MnO_2_/CNTs−80 fiber electrodes. The insets show the surface of the each fiber electrode. (scale bar = 500 µm and 20 µm, respectively.)

**
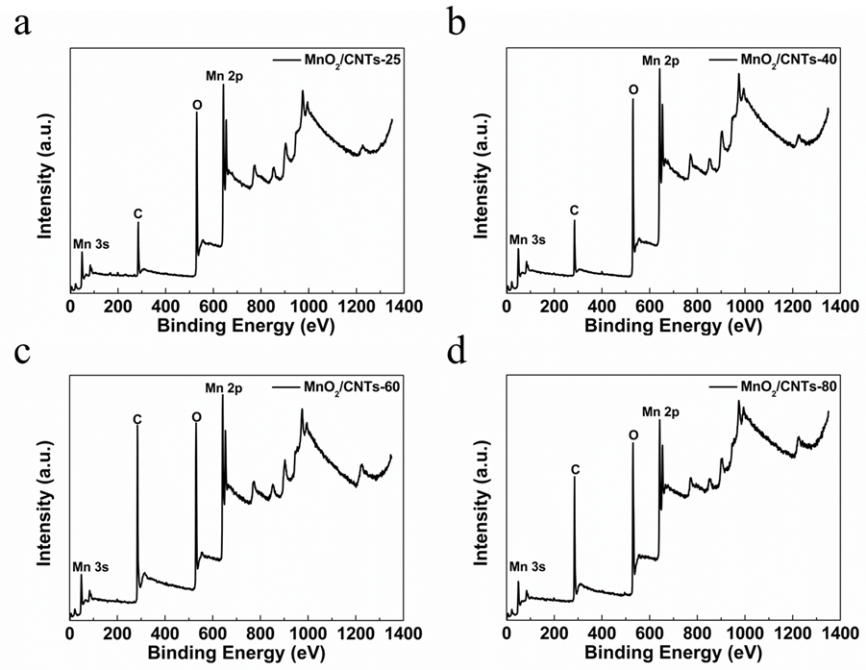
**

**Figure S2**. XPS survey spectra of MnO_2_/CNTs−25, MnO_2_/CNTs−40, MnO_2_/CNTs−60 and MnO_2_/CNTs−80 electrodes.


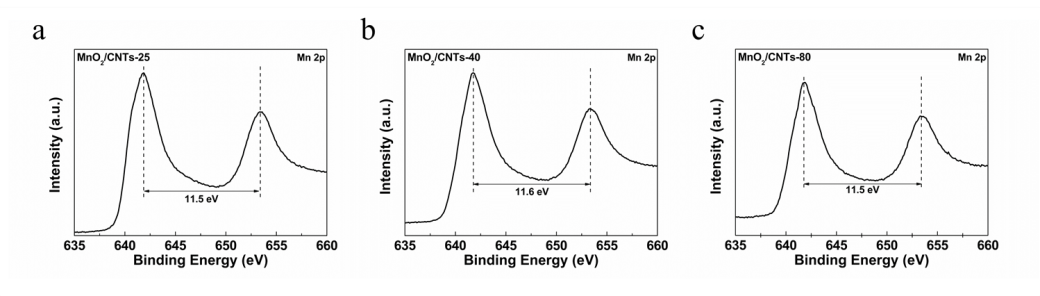


**Figure S3**. Mn 2p of XPS survey spectra of MnO_2_/CNTs−25, MnO_2_/CNTs−40, MnO_2_/CNTs−60 and MnO_2_/CNTs−80 electrodes.


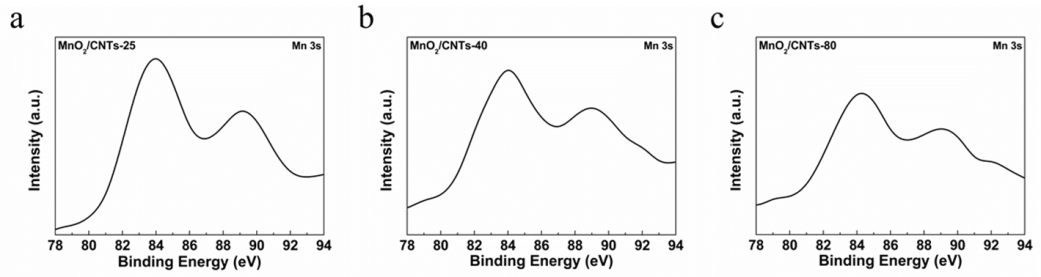


**Figure S4**. Mn 3s of XPS survey spectra of MnO_2_/CNTs−25, MnO_2_/CNTs−40, MnO_2_/CNTs−60 and MnO_2_/CNTs−80 electrodes.

**
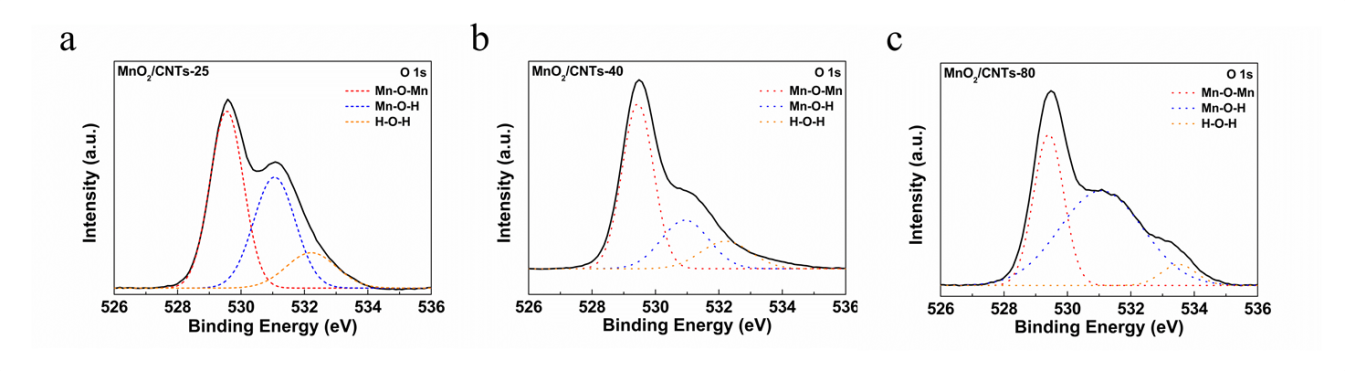
**

**Figure S5**. O 1s of XPS survey spectra of MnO_2_/CNTs−25, MnO_2_/CNTs−40, MnO_2_/CNTs−60 and MnO_2_/CNTs−80 electrodes.


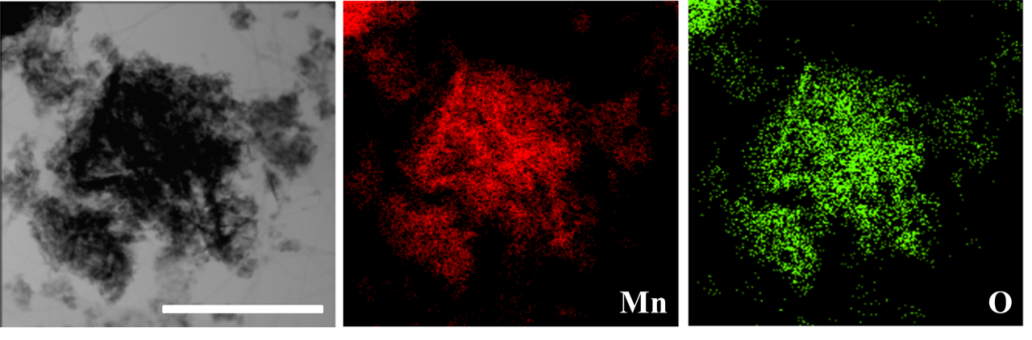


**Figure S6.** The TEM element mapping of MnO_2_ particles in the MnO_2_/CNTs−60 fiber electrodes.


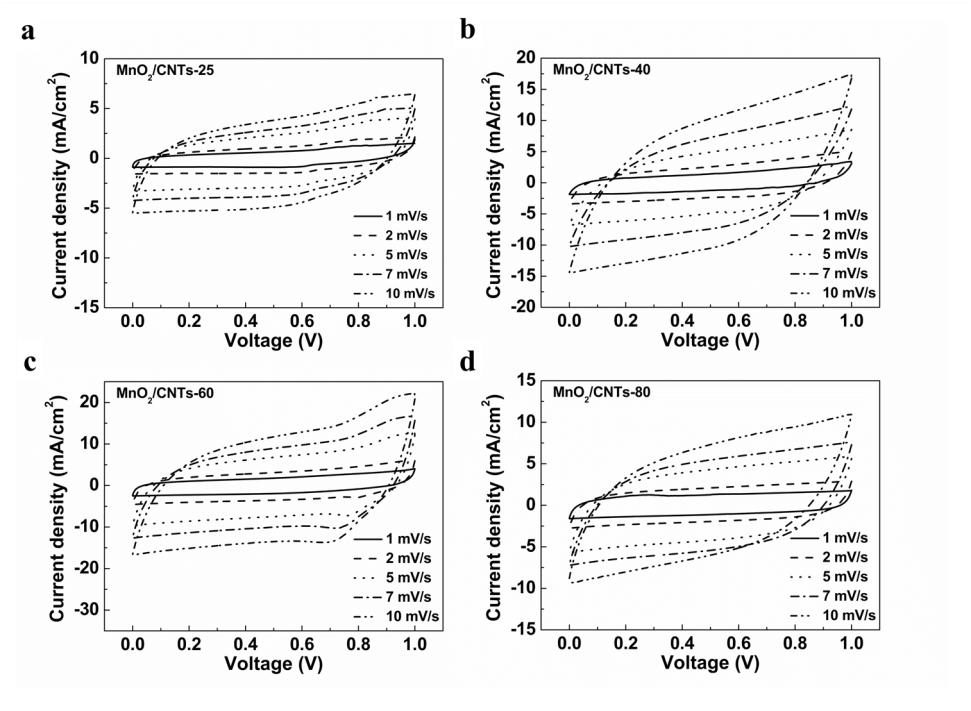


**Figure S7**. CV curves of MnO_2_/CNTs−25, MnO_2_/CNTs−40, MnO_2_/CNTs−60 and MnO_2_/CNTs−80 fiber electrodes at different scan rates.


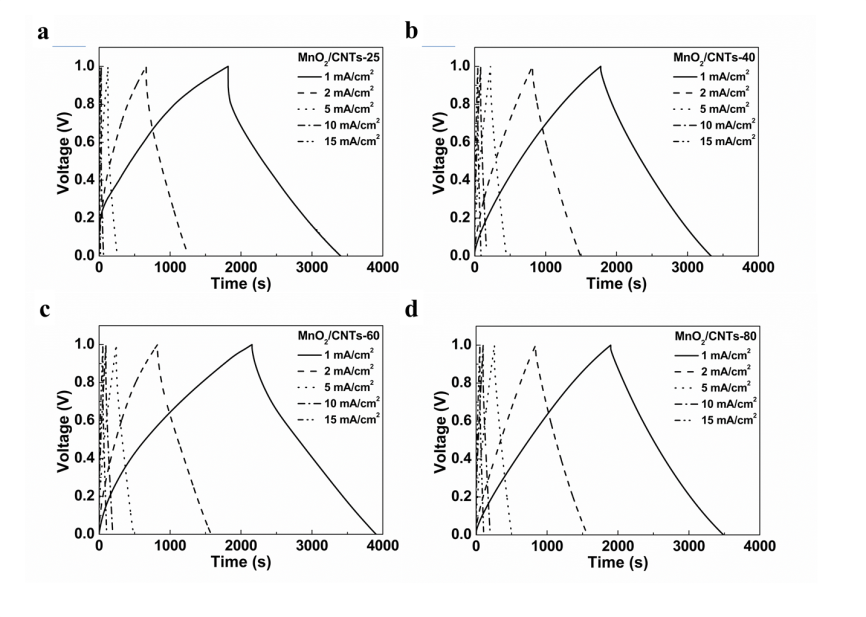


**Figure S8**. GCD profiles of MnO_2_/CNTs−25, MnO_2_/CNTs−40, MnO_2_/CNTs−60 and MnO_2_/CNTs−80 fiber electrodes at different current densities.

| **Sample Name** | **Capacitance : F/cm^2^ (F/cm^3^ and F/g)** | | | | | **Capacitance retention** |
| --- | --- | --- | --- | --- | --- | --- |
|  | 1 | 2 | 5 | 10 | 15 |  |
| **MnO_2_/CNTs−25** | 3.24 (445.9 and 292.1) | 2.42 (333.1 and 218.2) | 1.78 (245.3 and 160.6) | 1.24 (171.5 and 112.3) | 0.915 (126.1 and 82.6) | 28.2 % |
| **MnO_2_/CNTs−40** | 3.31 (441.9 and 300.8) | 2.92 (389.8 and 265.3) | 2.54 (338.3 and 230.2) | 2.14 (284.8 and 193.8) | 1.84 (246.5 and 167.8) | 55.6 % |
| **MnO_2_/CNTs−60** | 3.56 (557.6 and 321.3) | 3.14 (509.2 and 283.4) | 2.66 (431.4 and 240.1) | 2.33 (378.7 and 210.7) | 2.12 (344.7 and 191.8) | 59.6 % |
| **MnO_2_/CNTs−80** | 3.07 (350.4 and 276.6) | 2.85 (326.2 and 257.5) | 2.51 (286.2 and 225.9) | 2.01 (230.7 and 182.1) | 1.66 (189.4 and 149.6) | 54.1 % |

**Table S1**. Summary of the electrochemical performance of the MnO_2_/CNTs−25, MnO_2_/CNTs−40, MnO_2_/CNTs−60 and MnO_2_/CNTs−80 fiber electrodes.


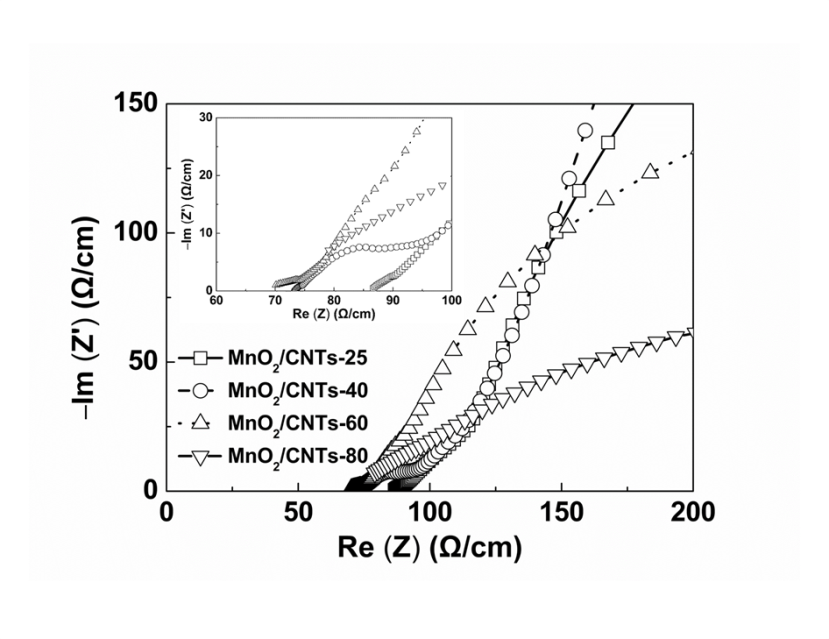


**Figure S9**. Nyquist plot of the MnO_2_/CNTs−25, MnO_2_/CNTs−40, MnO_2_/CNTs−60 and MnO_2_/CNTs-80 fiber electrodes. The inset shows higher resolution plots of the Nyquist curves.


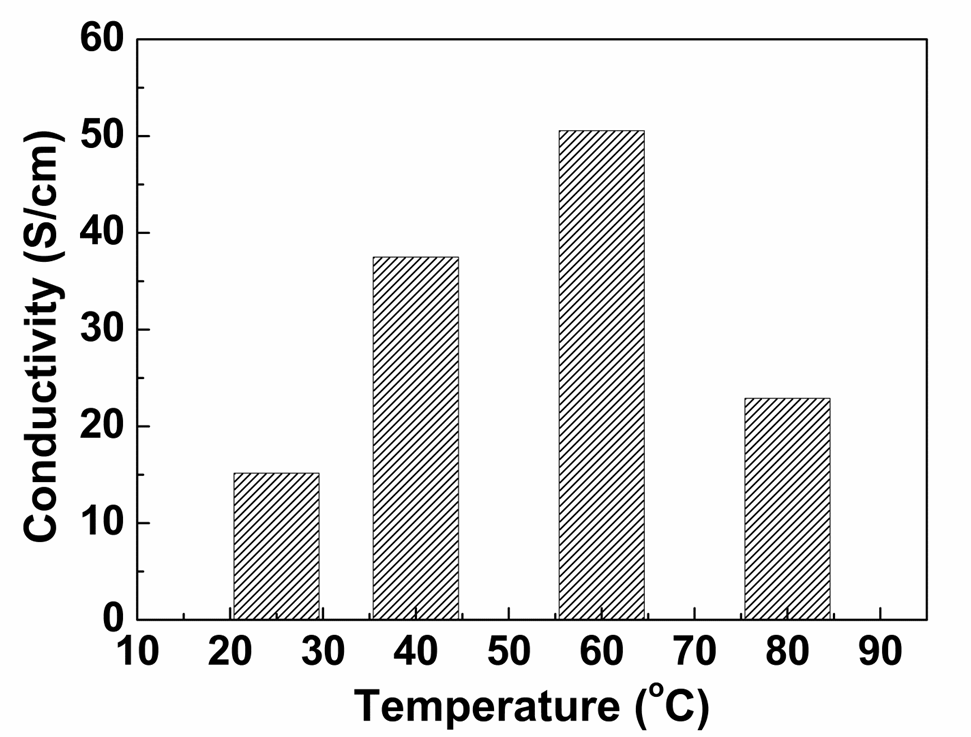


**Figure S10.** Conductivities of the MnO_2_/CNTs−25, MnO_2_/CNTs−40, MnO_2_/CNTs−60 and MnO_2_/CNTs−80 fiber electrodes.


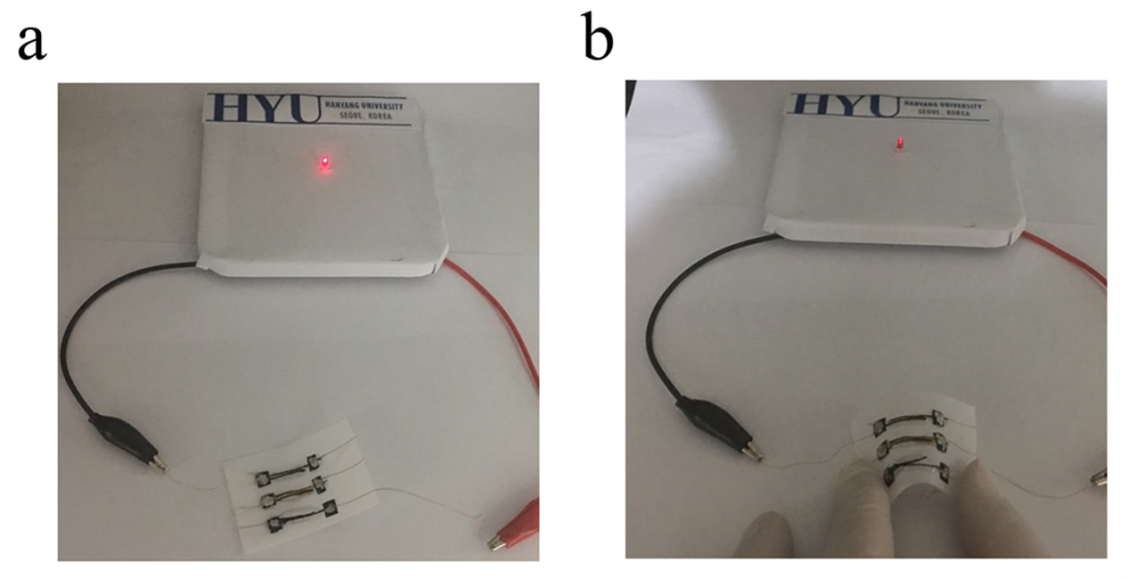


**Figure S11**. The optical images show that the red LED can be lighted with and without bending by three devices connected in series after charging.
